# Genetic Variation and Population Differentiation in the Bovine Lymphocyte Antigen *DRB3.2* Locus of South African Nguni Crossbred Cattle

**DOI:** 10.3390/ani11061651

**Published:** 2021-06-02

**Authors:** Lwamkelekile Sitshilelo Mkize, Oliver Tendayi Zishiri

**Affiliations:** School of Life Sciences, College of Agriculture, Engineering and Science, University of KwaZulu-Natal, Private Bag X54001, Durban 4000, South Africa; Zishiri@ukzn.ac.za

**Keywords:** allele, structure, disease resistance, genetic diversity, heterozygote

## Abstract

**Simple Summary:**

Indigenous cattle breeds are important to their native environment as they confer significant and often unique adaptability traits. The Nguni is one such breeds that is indigenous to the Southern African region. This breed confers resistance to thermal stress and diseases, amongst other factors. The bovine major histocompatibility complex is an important region, which codes for alleles that have been associated with a plethora of diseases. In the current study, the genetic diversity within this region was assessed in Nguni crossbred cattle. This was done to detect the gene pool of the Nguni breed, and to identify genes that might be important within this breed. The populations displayed a high degree of genetic diversity, and some alleles were common throughout the populations and accounted for a significant portion of the total alleles. This high genetic diversity could account for the great adaptability of the Nguni breed to Southern Africa.

**Abstract:**

The bovine lymphocyte antigen (*BoLA-DRB3*) gene is an important region that codes for glycoproteins responsible for the initiation of an immune response. *BoLA-DRB3* alleles have been demonstrated to be associated with disease resistance/tolerance. Therefore, great genetic diversity is correlated with better adaptation, fitness, and robustness. The current study was conducted to assess the population genetic structure of the *BoLA-DRB3* gene in Nguni crossbred cattle using polymerase chain reaction-sequence based typing (PCR-SBT). High genetic diversity was detected, with 30 alleles, 11 of which are novel to the study. Alleles DRB3*0201, DRB3*0701, DRB*0901, and DRB*1601 were present in all populations and accounted for nearly around 50% of all observed alleles. A mean genetic diversity (H_E_) of 0.93 was detected. The high overall genetic diversity is possibly associated with pathogen-assisted selection and heterozygote advantage. Such high diversity might explain the hardiness of the Nguni crossbred cattle to the Southern African region. Low population genetic structure was identified (F_ST_ = 0.01), suggesting possible gene flow between populations and retention of similar alleles. The study was undertaken to bridge the dearth of such studies in South African breeds and it is imperative for effective sustainability of indigenous breeds and the implementation of effective breeding strategies.

## 1. Introduction

The major histocompatibility complex (MHC) is one of the most important genetic regions in living organisms as it is ultimately responsible for the resistance or susceptibility to diseases [1]. Generally, the function of MHC molecules is to recognize and attach to pathogens/viruses and expose them to immune cells such as T-cells [2,3,4]. Defining the genetic variation in the MHC in different organisms indicates the degree to which populations can recognize foreign polypeptides. Hence, given this there is an induced propensity for individuals and populations to sustain genetic diversity, given that populations with greater genetic diversity at this region can recognize and bind a wider spectrum of pathogens [2]. Delineating the genetic architecture of the MHC is therefore of paramount importance in defining disease resistance and susceptibility.

In cattle this component is termed the bovine lymphocyte antigen (*BoLA*) and is located on chromosome 23 [5]. Within the BoLA region three *DRB* loci have been identified, namely, *DRB1*, *DRB2*, and *DRB3*, these genes encode the β-chain of the DR molecule [6]. The *DRB1* gene is a pseudogene comprised of multiple stops codons and low genetic diversity [6,7,8]. *DRB2* is a monomorphic region with very low expression levels [9,10] similar to *DRB1*, the *DRB2* gene is yet to be elucidated further. The most functional and highly expressed is the *DRB3* gene [11,12]. The second exon in the *BoLA-DRB3* is characterized with increased genetic variation and an even distribution of alleles [13]. This variation has been postulated to be maintained through different selective mechanisms [13,14]. A plethora of alleles have been uncovered and their association with various important livestock diseases such as ticks and tick-borne diseases [15,16], mastitis [17,18,19,20], and bovine leukemia virus [21,22,23], amongst others, have been studied. All the above studies revealed a high degree of genetic polymorphism in the *BoLA-DRB3* region. It is therefore ideal for populations to encompass significant variation in the MHC given that the greater the genetic diversity the greater the spectrum of pathogens that can be identified. Furthermore, this diversity is retained in populations due to various selective mechanisms such as pathogen-mediated selection. As a host’s immune system develops defensive mechanisms against a pathogen, the pathogen in turn develops defense systems and it is this arms race that will require the host to accumulate and retain genetic diversity [24].

Genetic diversity within the *BoLA-DRB3* region has been studied in many cattle breeds across the world. However, there is a paucity of studies that uncover the genetic basis of regions of immunological importance in African breeds. It is therefore imperative to define the genetic structure of indigenous breeds to improve their sustainability, as their adaptability to local environments has been created by long environmental selection. The Nguni is reared across most of Southern Africa, especially by smallholders [25]. Breeding systems in the smallholder sector are not usually amply defined and as such breeds like the Nguni risk losing their genetic structure due to crossing with exotic breeds [26]. Breed genetic diversity and its maintenance is crucial for adaptation trait selection. The main objective of the study was to detect the allele frequency distribution and overall genetic structure in South African Nguni crossbreds at the second exon of the *BoLA-DRB3* region. The Nguni were chosen due to their high numbers in the country and their adaptation to the natural environment of southern Africa. Against this background, the current study was conducted to fill the dearth in genetic information in the BoLA-DRB3 region of South African Nguni crossbred cattle. 

## 2. Materials and Methods

### 2.1. Studied Populations and DNA Extraction

**Figure 1:**
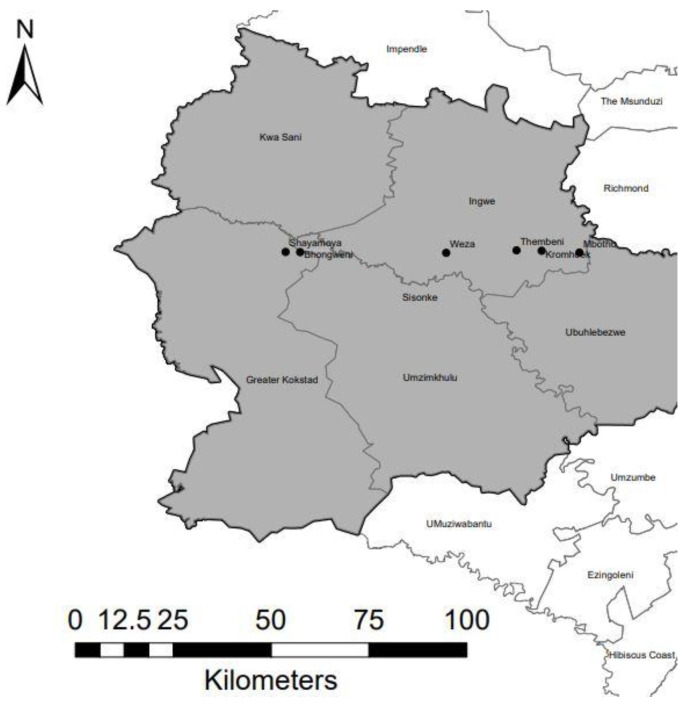
Map of areas where Nguni crossbred cattle were sampled (denoted by black circles) in the KwaZulu-Natal province of South Africa.

The study was approved by the Animal Research Ethics Committee of the University of Kwa-Zulu Natal (Reference numbers AREC/051/017M, AREC 071/017, AREC 014/018). The field sampling protocols, samples collected from animals, and the research were conducted in full compliance with Section 20 of the Animal Diseases Act of 1984 (Act No 35 of 1984) and were approved by the South African Department of Agriculture, Forestry and Fisheries DAFF (Section 20 approval reference number 12/11/1/5). Blood samples of 139 Nguni crossbreds were collected from six different populations. The different populations were defined based on their common shared geographical area, therefore, all individuals from the same population shared the same communal grazing area and they were allowed to interbreed. Furthermore, these populations were collected in two different districts, which are connected by a common road (R56). The sampled populations were Bhongweni = 22; Kromhoek = 24; Mbotho = 21; Shayamoya = 24; Thembeni = 24; and Weza = 24, which are within the province of KwaZulu-Natal, South Africa (Figure 1). Of the total studied animals, 87 were cows and 52 were either bulls or steers. 

Animals shared communal grazing land and were devoid of defined breeding systems. Some animals were herded on a daily basis due to high levels of theft in certain areas, however the majority were allowed to graze without any herdsmen. The majority of the animals in the sampled populations were Nguni crossbreds, and some were non-descript crossbreds. However, most people preferred the Nguni crossbred, and this was evident from the number of these crossbreds. The majority of breeds that were crossed with the Nguni were Angus, Brahman, and Bonsmara. However, due to lack of animal recording and defined breeding strategies, the exact contributing breeds resulting in the sampled composite could not be ascertained. 

Blood samples were collected from the tail of individuals at dipping facilities under the supervision of representatives of the Department of Agriculture in KwaZulu-Natal. Approximately 3 mL of blood was collected from the tail vein of each individual. The blood samples were stored in VACUETTE tubes coated with EDTA (Greiner Bio-One, Kremsmuenster, Austria) with immediate storage on ice. Genomic DNA was extracted from each sample using the Quick-gDNA Blood Miniprep (Zymo Research, Orange, CA, USA) following the manufacturer’s instructions. Sampling was done with the aid of local herdsmen to avoid sampling related individuals within the same herd; hence all study individuals were presumed to be unrelated.

### 2.2. Polymerase Chain Reaction-Sequence Based Typing (PCR-SBT) of BoLA-DRB3

The second exon of the *BoLA-DRB3* locus was amplified as described by van Eijk et al. [27], with some modifications. The first PCR reaction was conducted in a total volume of 25 µL containing 1× DreamTaq Green PCR Mastermix (Thermo Scientific, Waltham, MA, USA), 0.5 µM of each of the two primers; HLO30 5′-ATCCTCTCTCTGCAGCACATTTCC-3′ and HLO31 5′-TTTAAATTCGCGCTCACCTCGCCGCT-3, and 20 ng of template DNA. Thermal cycling was conducted in a T100™ Thermal Cycler (BIO RAD, Richmond, CA, USA) employing the following conditions; 94 °C for 4 min, 15 repeats (94 °C for 1 min, 60 °C for 2 min, 72 °C for 90 s), and a final extension step of 72 °C for 5 min.

The second amplification round was also conducted in total volume of 25 µL containing 1× DreamTaq Green PCR Mastermix (Thermo Scientific, Waltham, MA, USA), 0.5 µM of each of the two primers; HLO30 5′-ATCCTCTCTCTGCAGCACATTTCC-3′ and HLO32 5′-TCGCCGCTGCACAGTGAAACTCTC-3′, and 2 µL of the PCR products of the first round. Thermal cycling was employed using the following conditions; 40 repeats of 94 °C for 1 min and 65 °C for 30 s and a final extension step of 72 °C for 5 min. The PCR amplicons were then separated by gel electrophoresis in a 1% agarose gel and viewed under ultraviolet illumination after staining with ethidium bromide. Thereafter, the PCR products were cleaned by mixing 10 µL of the PCR product with 2.5 µL of Exo/SAP mix; exonuclease 1 (NEB M0293) 20 U/µL; Shrimp alkaline phosphatase (NEB M0371) 1 U/µL. The mixture was incubated at 37 °C for 30 min then the reaction was stopped by heating the mixture at 95 °C for 5 min. DNA sequencing was then conducted using the ABI V3.1 Big dye kit (Applied Biosystems, Foster City, CA, USA) in accordance with the manufacturer’s instructions on an ABI 377 automated sequencer (Applied Biosystems, Foster City, CA, USA). The Zymo Seq clean-up kit (Zymo Research, Orange, CA, USA) was used to clean the labelled products thereafter, the products were injected in the ABI 3500XL Genetic Analyzer (Applied Biosystems, Foster City, CA, USA) using POP7. A representation of the sequences that emanated from the South African crossbred Nguni cattle from our study were deposited in GenBank with accession numbers MN721876 to MN721885.

### 2.3. Population Genetic Diversity

Number of alleles (NA) and allele frequencies were obtained through direct counting, the formula employed by Das et al. [11] was utilized for determining the allele frequencies. Furthermore, POPGENE 1.3 [28] was used to validate the number of alleles, allele frequencies and to estimate the number of effective alleles (NE) per population [29]. Observed (HO) and expected heterozygosity (HE) was assessed following Nei [30], using ARLEQUIN 3.5 [31]. Genotypic departure from Hardy-Weinberg equilibrium (HWE) across all populations was determined by the FIS [32] statistic using an exact test in GENEPOP 4.0 [33] and significance was tested at 0.05 using the Markov chain algorithm [34]. 

### 2.4. Population Genetic Structure and Differentiation

Genetic partitioning among and within populations was detected by means of analysis of molecular variance (AMOVA) utilizing 1000 permutations in ARLEQUIN 3.5. Genetic differentiation was evaluated using exact tests [35] employing 100,000 Markov chains. Pairwise FST and Slatkins linearized FST indices were evaluated using ARLEQUIN 3.5. The functions pairFstMatrix.r and slatkinFstFunction.r in R were used to generate a graphical representation of the FST estimates. 

## 3. Results

### 3.1. Allelic Diversity and Frequency

A total of 30 alleles were identified in the studied population (Table 1). Of those alleles, 19 have already been previously identified and 11 are unreported. These new alleles were present in at least two populations each with overall frequency per allele ranging from 0.0072 (DRB3*10) to 0.0288 (DRB*8) and contributing 18% of the total alleles in the overall studied population. Alleles DRB3*0201, DRB3*0701, DRB*0901, and DRB*1601 were present in all populations and accounted for nearly around 50% of all observed alleles in the populations. DRB3*0901 was the most frequent in the study with a frequency of 0.144, and DRB3*03021 was only detected in the Shayamoya population with moderate frequency (>0.5). 

### 3.2. Population Genetic Diversity

Genetic diversity estimates among the populations depicted that the number of alleles ranged from 12 (Mbotho) to 19 (Bhongweni; Table 2). The Mbotho population also contained the least number of new alleles, whereas the Bhongweni and Kromhoek populations contained 7 new alleles each, which were more than other populations. Of the new alleles only two, DRB3*1 and DRB3*8, were identified with a frequency greater than 0.5 in the Bhongweni and Thembeni populations, respectively. Expected heterozygosity ranged from 0.882 for Mbotho to 0.941 for Bhongweni (Table 2). The observed heterozygosity ranged from 0.833 (Shayamoya and Weza) to 0.961 (Thembeni). Interestingly, the Mbotho population contained the least number of alleles however it displayed the great genetic distribution. Moreover, even though the Bhongweni population contained the greatest number of alleles, it lacked genetic distribution and was the third lowest population in terms of observed heterozygosity. Three populations, Bhongweni, Shayamoya, and Weza, displayed partial excess of homozygotes, however they did not deviate from HWE. Furthermore, Kromhoek, Mbotho, and Thembeni displayed minor heterozygote excess and all but Mbotho did not deviate from HWE. 

### 3.3. Genetic Structure and Differentiation

**Figure 2:**
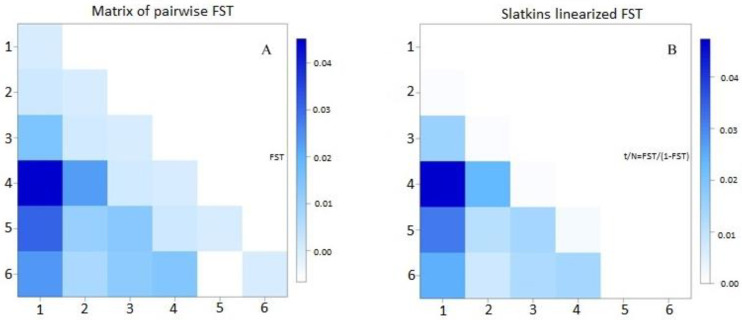
Graphical representation of (**A**) pairwise F_ST_ and (**B**) Slatkins linearized F_ST_ estimates between 6 Nguni crossbred populations based on the *BoLA-DRB3* gene. 1 = Bhongweni, 2 = Kromhoek, 3 = Mbotho, 4 = Shayamoya, 5 = Thembeni, 6 = Weza.

The genetic diversity was partitioned into two sources of variation, viz. variation among populations and variation within populations, in an AMOVA. It was revealed that the majority (99%) of the genetic variation can be explained by genetic diversity within individuals in the populations and only 1% of the variation can be explained by genetic differences amongst populations (Table 3). Limited genetic sub-structuring between populations revealed in the AMOVA can be further validated by the average pairwise F_ST_ value of 0.011 (Table 4 and Figure 2A) and Slatkins linearized F_ST_ of 0.014 (Table 4 and Figure 2B). Of all the 15 population pairwise F_ST_ values, estimates ranged from 0 (between Kromhoek and Mbotho; Shayamoya and Thembeni; Thembeni and Weza) to 3.3% (between Bhongweni and Shayamoya), and Slatkins F_ST_ indices ranged from 0 to 4.7% for the same population pairs. 

## 4. Discussion

Exon 2 of the *BoLA-DRB3* gene is highly important and plays a crucial role in the response of T-cells to pathogens [36]. To date there has been 303 defined alleles in this region (https://www.ebi.ac.uk/ipd/mhc/group/BoLA/, accessed on 3 March 2021). Hence, substantial genetic variation in this region could account for the association between efficient immune response and disease resistance [37,38]. The current study reveals that South African crossbred Nguni cattle exhibits high genetic polymorphism in the *BoLA-DRB3* region. 

The Nguni is a breed that is very common in South Africa. It was introduced into southern Africa by way of three migrations by pastoralists [39]. It is widespread and possesses superior qualities such as tick resistance [40,41], and has the ability to survive in remote rural areas of low input systems where the quality of forages is poor [41,42]. Nguni cattle have also been revealed to produce ample milk [43]. A significant number of communal farmers in South Africa keep the Nguni crossbreed, given this the genetic diversity of such a breed and others needs to be unpacked. The studied population revealed 30 alleles which is consistent with 21 alleles detected in Japanese Shorthorn cattle [44], 38 in Hanwoo cattle [45], 35 in Yacumeño cattle [46], and 33 in Nellore cross Brahman cattle [47]. Alleles DRB3*0201, DRB3*0701, DRB*0901, and DRB*1601 were found to be present in all the studied populations. Several studies have been conducted to assess possible associations between DRB3 alleles and disease resistance, they have been shown to be significantly correlated with CD4, CD8, and CD12 cells [1]. Mastitis resistance has been found to be associated with alleles *0201, *0901 [48,49]. Furthermore, the *0901 allele has demonstrated an association with resistance to bovine leukemia virus (BLV) in different regions [21,50]. The alleviation of foot-and-mouth disease (FMD) was associated with allele *0201 amongst others in Hereford steers [38] and allele *1601 has shown a response to FMD vaccines [51]. The two alleles *0201 and *0901 have been illustrated to be associated with several diseases in different species of cattle. This suggests that these alleles play an important role in disease resistance. All populations in the current study possessed high frequencies of both alleles. Further validation studies across the spectrum of African cattle breeds across different countries need to be conducted and possible marker assisted selection to improve disease resistance in cattle. 

There was favorable genetic distribution within populations as this was evident from the high levels of observed heterozygosity. Three of the six populations (Kromhoek, Mbotho, and Thembeni) display an excess of heterozygotes and partial outbreeding. However, no population displayed significant heterozygote excess or deficiency and did not violate HWE. Heterozygote advantage (overdominance) or recent bottleneck events that results in reduced effective population sizes [52] could be plausible explanations for this high heterozygosity. When heterozygotes confer greater fitness at a locus, selection will tend to favor heterozygote genotypes and this will result in reduction of homozygotes and subsequent increase in heterozygotes [53]. Pathogen-mediated selection could also contribute to heterozygote retention and advantage because pathogens gradually confer resistance to the defense mechanisms of hosts, subsequently the host will oppose this by accumulation and retention of genetic variation [24]. The MHC is a region that is highly endowed in genetic diversity in natural populations [54,55,56]. This region is involved in production of glycoproteins that bind to foreign bodies and divert them to crucial components of the immune system [57]. Thus, in theory a greater array of alleles would allow individuals to produce different glycoproteins, enabling them to identify a plethora of pathogens. Furthermore, support of this has been illustrated in a study in mice whereby mice heterozygous at the MHC displayed greater resistance to multiple-strain infections of Salmonella [14]. Parallel results were reported by Worley et al. [58] in junglefowl where it was revealed that individuals with heterozygous genotypes for the MHC survived coccidiosis infection longer than individuals with homozygous genotypes. Therefore, the Nguni populations in this study could be resistant to several diseases given the high genetic diversity revealed. 

The BoLA-DRB3.2 locus is defined by a low population structure in South African Nguni cattle. Pairwise F_ST_ indices depicted very limited genetic difference between populations, these estimates ranged from 0 (between Thembeni and Weza) to 3.3% (between Bhongweni and Shayamoya). They were comparable to genetic differentiation (F_ST_ = 0.009) identified in Holstein cattle [59], albeit the Holstein study covered samples from four different South American countries. This, therefore, depicts the retention of important alleles in cattle, or the high degree of inbreeding in the Holstein breed [60,61]. The population differentiation estimates in the current study were, however, lower than those reported for Mexican creole breeds [4]. Furthermore, Zebu cattle from South America revealed an FST range between 2.2% and 13.3% [47], which is much greater than that of our studied populations. This could be due to the diverse breeds used in the South American study [42], which studied this diversity in three different breeds.

The use of adaptive/non-neutral markers, such as the BoLA-DRB3 gene in this study, is vital in studying the population genetics and the extent of local adaptations in species [62]. Nonetheless, neutral genetic markers have been the most widely used tools due to their ease of availability and because they are unbiased in assessing some genetic parameters [63]. On the contrary, given the ever-changing environment and plasticity of species, non-neutral markers are optimal as populations adapt to environmental stressors due to these non-neutral markers [64]. The use of neutral markers in the current study would have possibly revealed similar results in terms of population differentiation. This is because neutral genetic markers are ideal for inferring spatial genetic structure and gene flow [65], and given the cultural practices and proximity of the studied areas, there should be an ample amount of gene flow among populations. Neutral genetic markers would, however, show an increase in genetic diversity indicis, compared to the non-neutral marker used in the current study. This is because neutral markers are not subjected to natural selection [62], and therefore should evolve more rapidly than non-neutral markers. 

An analysis of molecular variance depicted that the majority of genetic variance (99%) was partitioned within populations and only 1% of the genetic variance was between populations. This depicts an even allele distribution between populations. The low population genetic differentiation returned can be explained by environmental and pathogen-mediated selection (as already explained above), whereby similar alleles will be maintained in populations due to their relative fitness, and since these populations belong to the same province environmental conditions and other stressors are expected to be somewhat constant across populations hence possibly enabling the retention of similar alleles. Gene flow may also be shaping the genetic structure of the current populations, it is common for farmers to exchange cattle between different regions given the accessibility of the study areas. 

## 5. Conclusions

Great genetic diversity was detected with 11 novel alleles identified. This high genetic diversity could possibly be maintained by a heterozygote advantage (overdominance). Low population differentiation was returned, which points to the retention of similar alleles between populations. Such information is crucial for defining the gene pool of indigenous breeds and outlining the genetic basis of their adaptation to local environments. When the genetic architecture of the *BoLA* is amply illuminated association studies of immunological traits can be assessed and sustainable breeding systems can be put into place.

## Figures and Tables

**Table 1:** Gene frequencies of *BoLA-DRB3* gene in Nguni crossbred cattle populations from KwaZulu-Natal, South Africa.

Allele	Bhongweni	Kromhoek	Mbotho	Shayamoya	Thembeni	Weza	Overall
DRB3*0101		0.0208	0.0476	0.0625	0.0208	0.0208	0.0288
DRB3*0201	0.0455	0.1042	**0.2143**	**0.2083**	0.1458	0.0833	0.1331
DRB3*03021				0.0625			0.0108
DRB3*0501	0.0909	0.0417		0.0208	0.0417	0.0417	0.0396
DRB3*0701	**0.1591**	0.1042	0.1429	0.0417	0.0208	0.0833	0.0899
DRB3*0901	0.1364	**0.1875**	**0.2143**	0.1042	0.1042	0.1250	**0.1439**
DRB3*1001	0.0227	0.1042		0.0417	0.0417		0.0360
DRB3*1002	0.0682					0.0208	0.0144
DRB3*1103			0.0476	0.0417	0.0417		0.0216
DRB3*1201		0.0417			0.0208	0.0208	0.0144
DRB3*1501	0.0455	0.0208		0.0208	0.0417		0.0216
DRB3*1601	0.0227	0.0625	0.1190	0.1667	**0.2083**	**0.1875**	0.1295
DRB3*1801	0.0227	0.0208	0.0476			0.0417	0.0216
DRB3*20012		0.0417	0.0238		0.0208	0.0417	0.0216
DRB3*2801		0.0625	0.0238		0.0417	0.0625	0.0324
DRB3*3001	0.0227				0.0417		0.0108
DRB3*3101				0.0625	0.0208	0.0625	0.0252
DRB3*3202	0.0227	0.0208				0.0208	0.0108
DRB3*4201	0.0682			0.0208			0.0144
DRB3*1	0.0682		0.0238				0.0144
DRB3*2	0.0227	0.0208			0.0208		0.0108
DRB3*3	0.0455	0.0417	0.0476			0.0208	0.0252
DRB3*4	0.0227	0.0208			0.0417		0.0144
DRB3*5		0.0208		0.0417	0.0417	0.0417	0.0252
DRB3*6		0.0208				0.0417	0.0108
DRB3*7		0.0208				0.0417	0.0108
DRB3*8	0.0455				0.0833	0.0417	0.0288
DRB3*9	0.0455			0.0417			0.0144
DRB3*10	0.0227			0.0208			0.0072
DRB3*11		0.0208		0.0417			0.0180

New alleles are underlined; alleles with greatest frequencies per population are in bold.

**Table 2:** Descriptive genetic parameters in *BoLA-DRB3* gene of six Nguni crossbred populations from KwaZulu-Natal, South Africa.

Population	N	N_A_	N_E_	N_NA_	N_NA_ > 0.5	H_O_	H_E_	F_IS_
Bhongweni	22	19	12.41	7	1	0.864	0.941	0.0607 ^NS^
Kromhoek	24	20	11.52	7	0	0.958	0.933	−0.0494 ^NS^
Mbotho	21	12	7.17	2	0	0.947	0.882	−0.0737 ^NS^
Shayamoya	24	16	9.44	4	0	0.833	0.913	0.0680 ^NS^
Thembeni	24	18	10.11	4	1	0.961	0.920	−0.0636 ^NS^
Weza	24	18	11.52	5	0	0.833	0.933	0.0875 ^NS^
Overall	139	30	13.35	11	0	0.899	0.928	0.0049

N, number of individuals per population; N_A_, number of alleles; N_E_, number of effective alleles; N_NA_, number of newly identified alleles; N_NA_ > 0.5, number of newly identified alleles with frequency greater than 5%. H_O_, observed heterozygosity; H_E_, expected heterozygosity; F_IS_, inbreeding coefficient; NS, non-significant (*p* > 0.05).

**Table 3:** Genetic partitioning of genetic diversity in *BoLA-DRB3* gene among six Nguni crossbred populations of KwaZulu-Natal, South Africa.

Source of Variation	Sum of Squares	Variance Components	Percentage Variation
Among Populations	3.371	0.00462	0.96315
Within Populations	125.215	0.46035	99.03685
Total	128.586	0.46497	

**Table 4:** Pairwise F_ST_ estimates (below diagonal) and Slatkins linearized F_ST_ estimates (above diagonal) in *BoLA-DRB3* gene between six Nguni crossbred populations of KwaZulu-Natal, South Africa.

Sampling Area	Bhongweni	Kromhoek	Mbotho	Shayamoya	Thembeni	Weza
Bhongweni	-	0.00143	0.01534	0.04728	0.03171	0.02449
Kromhoek	0.00437	-	0.00100	0.02352	0.01056	0.00779
Mbotho	0.02097	0.00064	-	0.00087	0.01339	0.01214
Shayamoya	0.03278	0.01332	0.00324	-	0.00174	0.01379
Thembeni	0.02907	0.01045	0.01448	0.00161	-	0
Weza	0.01625	0.00150	0.00821	0.00454	0	-

## Data Availability

A representation of the sequences that emanated from the study were deposited in GenBank with accession numbers MN721876 to MN721885.

## References

[1] Brujeni G.N., Ghorbanpour R., Esmailnejad A. (2016). Association of BoLA-DRB3.2 Alleles with BLV Infection Profiles (Persistent Lymphocytosis/Lymphosarcoma) and Lymphocyte Subsets in Iranian Holstein Cattle. Biochem. Genet..

[2] Piertney S.B., Oliver M.K. (2005). The evolutionary ecology of the major histocompatibility complex. Heredity.

[3] Adams E.J., Luoma A.M. (2013). The Adaptable Major Histocompatibility Complex (MHC) Fold: Structure and Function of Nonclassical and MHC Class I–Like Molecules. Annu. Rev. Immunol..

[4] Fernández I., Leyva-Baca I., Rodriguez-Almeida F., Ulloa-Arvizu R., Ríos-Ramírez J., Gayosso-Vázquez A., Alonso-Morales R. (2015). Creole cattle from northwestern Mexico has high genetic diversity in the locus DRB3.2. Anim. Genet. Resour. Ressour. Génétiques Anim. Recur. Genéticos Anim..

[5] Fries R., Hediger R., Stranzinger G. (2009). Tentative chromosomal localization of the bovine major histocompatibility complex by in situ hybridization. Anim. Genet..

[6] Behl J.D., Verma N.K., Tyagi N., Mishra P., Behl R., Joshi B.K. (2012). The Major Histocompatibility Complex in Bovines: A Review. Int. Sch. Res. Not..

[7] Groenen M.A., Van Der Poel J.J., Dijkhof R.J., Giphart M.J. (1990). The nucleotide sequence of bovine MHC class II DQB and DRB genes. Immunogenetics.

[8] Ellis S.A., Ballingall K.T. (1999). Cattle MHC: Evolution in action?. Immunol. Rev..

[9] Burke M.G., Stone R.T., Muggli-Cockett N.E. (2009). Nucleotide sequence and Northern analysis of a bovine major histocompatibility class II DRß-like cDNA. Anim. Genet..

[10] Konnai S., Nagaoka Y., Takesima S., Onuma M., Aida Y. (2003). Technical Note: DNA Typing for Ovine MHC DRB1 Using Polymerase Chain Reaction-Restriction Fragment Length Polymorphism (PCR-RFLP). J. Dairy Sci..

[11] Das D.N., Sri Hari V.G., Hatkar D.N., Rengarajan K., Saravanan R., Suryanarayana V.V., Murthy L.K. (2012). Genetic diversity and population genetic analysis of bovine MHC class II DRB3. 2 locus in three Bos indicus cattle breeds of Southern India. Int. J. Immunogenet..

[12] Giovambattista G., Moe K.K., Polat M., Borjigin L., Hein S.T., Moe H.H., Takeshima S.-N., Aida Y. (2020). Characterization of bovine MHC DRB3 diversity in global cattle breeds, with a focus on cattle in Myanmar. BMC Genet..

[13] Goszczynski D.E., Ripoli M.V., Takeshima S., Baltian L., Aida Y., Giovambattista G. (2014). Haplotype determination of the upstream regulatory region and the second exon of the BoLA-DRB3 gene in Holstein cattle. Tissue Antigens.

[14] Penn D.J., Damjanovich K., Potts W.K. (2002). MHC heterozygosity confers a selective advantage against multiple-strain infections. Proc. Natl. Acad. Sci.USA.

[15] Martinez M.L., Machado M.A., Nascimento C.S., Silva M.V., Teodoro R.L., Furlong J., Prata M.C., Campos A.L., Guimarães M.F., Azevedo A.L. (2006). Association of BoLA-DRB3. 2 alleles with tick (*Boophilus microplus*) resistance in cattle. Genet. Mol. Res..

[16] Duangjinda M., Jindatajak Y., Tipvong W., Sriwarothai J., Pattarajinda V., Katawatin S., Boonkum W. (2013). Association of BoLA-DRB3 alleles with tick-borne disease tolerance in dairy cattle in a tropical environment. Vet. Parasitol..

[17] Firouzamandi M., Shoja J., Barzegari A., Roshani E. (2010). Study on the association of BoLA-DRB3. 2 alleles with clinical mastitis in Iranian Holstein and Sarabi (Iranian native) cattle. Afr. J. Biotechnol..

[18] Zambrano J.C., Echeverri J., López-Herrera A. (2011). Alleles of the BoLA DRB3. 2 gene are associated with mastitis in dairy cows. Rev. Colomb. Cienc. Pecu..

[19] Ramírez N.F., Montoya A., Cerón-Muñoz M.F., Villar D., Palacio L.G. (2014). Association of BoLA-DRB3 and TLR4 alleles with subclinical mastitis in cattle from Colombia. Rev. Colomb. Cienc. Pecu..

[20] Suprovych T.M., Suprovych M.P., Koval T.V., Karchevska T.M., Chepurna V.A., Chornyi I.O., Berezhanskyi A.P. (2018). BoLA-DRB3 gene as a marker of susceptibility and resistance of the Ukrainian black-pied and red-pied dairy breeds to mastitis. Regul. Mech. Biosyst..

[21] Panei C., Suzuki K., Echeverria M., Serena M., Metz G.E., Gonzalez E. (2009). Association of BoLA-DRB3.2 Alleles with Resistance and Susceptibility to Persistent Lymphocytosis in BLV Infected Cattle in Argentina. Int. J. Dairy Sci..

[22] Mosafer J., Heydarpour M., Manshad E., Russell G., Sulimova G.E. (2012). Distribution of BoLA-DRB3 allelic frequencies and identification of two new alleles in Iranian Buffalo breed. Sci. World J..

[23] Farias M.V.N., Caffaro M.E., Lendez P.A., Passucci J., Poli M., Ceriani M.C., Dolcini G.L. (2017). A novel association of BoLA DRB3 alleles in BLV infected cattle with different proviral loads. Braz. J. Vet. Res. Anim. Sci..

[24] Jeffery K.J., Bangham C.R. (2000). Do infectious diseases drive MHC diversity?. Microbes Infect..

[25] Mapiye O., Chikwanha O.C., Makombe G., Dzama K., Mapiye C. (2020). Livelihood, Food and Nutrition Security in Southern Africa: What Role Do Indigenous Cattle Genetic Resources Play?. Divers..

[26] Khombe C.T. (2002). Genetic Improvement of Indigenous Cattle Breeds in Zimbabwe: A Case Study of the Mashona Group Breeding Scheme.

[27] van Eijk M.J.T., Stewart-Haynes J.A., Lewin H.A. (1992). Extensive polymorphism of the BoLA-DRB3 gene distinguished by PCR-RFLP. Anim. Genet..

[28] Yeh F.C., Yang R.C., Boyle T.B., Ye Z.H., Mao J.X. (1997). The User-Friendly Shareware for Population GENETIC analysis. Mol. Biol. Biotechnol. Cent. Univ. Alta. Can..

[29] Kimura M., Crow J.F. (1964). The number of alleles that can be maintained in a finite population. Genetics.

[30] Nei M. (1978). Estimation of Average Heterozygosity and Genetic Distance from a Small Number of Individuals. Genetics.

[31] Excoffier L., Laval G., Schneider S. (2005). Arlequin (version 3.0): An integrated software package for population genetics data analysis. Evol. Bioinform. Online.

[32] Weir B.S., Cockerham C.C. (1984). Estimating F-statistics for the analysis of population structure. Evolution.

[33] Rousset F. (2008). genepop’007: A complete re-implementation of the genepop software for Windows and Linux. Mol. Ecol. Resour..

[34] Guo S.W., Thompson E.A. (1992). Performing the Exact Test of Hardy-Weinberg Proportion for Multiple Alleles. Biometrics.

[35] Raymond M., Rousset F. (1995). An exact test for population differentiation. Evolution.

[36] Baxter R., Hastings N., Law A., Glass E.J. (2008). A rapid and robust sequence-based genotyping method for BoLA-DRB3 alleles in large numbers of heterozygous cattle. Anim. Genet..

[37] García-Briones M.M., Russell G.C.A., Oliver R., Tami C., Taboga O., Carrillo E., Palma E.L., Sobrino F., Glass E.J. (2000). Association of bovine DRB3 alleles with immune response to FMDV peptides and protection against viral challenge. Vaccine.

[38] Paracha H., Hussain T., Tahir M.Z., Yasmeen A., Pervez M.T., Sheikh A.A., Haider A., Ali R., Khan W.A. (2015). Multifunctional DRB3, a MHC class II gene, as a useful biomarker in small ruminants: A review. J. Infect. Mol. Biol..

[39] Ramsay K.A. (1988). Phenotypic Selection of the Nguni Cattle in Different Environments in Southern Africa.

[40] Marufu M., Chimonyo M., Dzama K., Mapiye C. (2010). Seroprevalence of tick-borne diseases in communal cattle reared on sweet and sour rangelands in a semi-arid area of South Africa. Vet. J..

[41] Marufu M., Qokweni L., Chimonyo M., Dzama K. (2011). Relationships between tick counts and coat characteristics in Nguni and Bonsmara cattle reared on semiarid rangelands in South Africa. Ticks Tick-Borne Dis..

[42] Mapekula M., Chimonyo M., Mapiye C., Dzama K. (2011). Fatty acid, amino acid and mineral composition of milk from Nguni and local crossbred cows in South Africa. J. Food Compos. Anal..

[43] Moyo S. (1996). The Productivity of Indigenous and Exotic Beef Breeds and Their Crosses at Matopos, Zimbabwe. Ph.D. Thesis.

[44] Takeshima S., Nakai Y., Ohta M., Aida Y. (2002). Short Communication: Characterization of DRB3 Alleles in the MHC of Japanese Shorthorn Cattle by Polymerase Chain Reaction-Sequence-Based Typing. J. Dairy Sci..

[45] Lee B.-Y., Hur T.-Y., Jung Y.-H., Kim H. (2011). Identification of BoLA-DRB3.2 alleles in Korean native cattle (Hanwoo) and Holstein populations using a next generation sequencer. Anim. Genet..

[46] Giovambattista G., Takeshima S.-N., Ripoli M.V., Matsumoto Y., Franco L.A.A., Saito H., Onuma M., Aida Y. (2013). Characterization of bovine MHC DRB3 diversity in Latin American Creole cattle breeds. Gene.

[47] Takeshima S.N., Corbi-Botto C., Giovambattista G., Aida Y. (2018). Genetic diversity of BoLA-DRB3 in South American Zebu cattle populations. BMC Genet..

[48] Dietz A.B., Cohen N.D., Timms L., Kehrli M.E. (1997). Bovine lymphocyte antigen class II alleles as risk factors for high somatic cell counts in milk of lactating dairy cows. J. Dairy Sci..

[49] Kulberg S., Heringstad B., Guttersrud O., Olsaker I. (2007). Study on the association of BoLA-DRB3.2 alleles with clinical mastitis in Norwegian Red cows. J. Anim. Breed. Genet..

[50] Lewin H. (1994). Host genetic mechanism of resistance and susceptibility to a bovine retroviral infection. Anim. Biotechnol..

[51] Baxter R., Craigmile S.C., Haley C., Douglas A.J., Williams J.L., Glass E.J. (2009). BoLA-DR peptide binding pockets are fundamental for foot-and-mouth disease virus vaccine design in cattle. Vaccine.

[52] Stilwell K.L., Wilbur H.M., Werth C.R., Taylor D.R. (2003). Heterozygote advantage in the American chestnut, Castanea dentata (Fagaceae). Am. J. Bot..

[53] Hamilton M.B. (2009). Population Genetics.

[54] Hughes M.K., Hughes A.L. (1995). Natural selection on Plasmodium surface proteins. Mol. Biochem. Parasitol..

[55] Apanius V., Penn D., Slev P.R., Ruff L.R., Potts W.K. (1997). The Nature of Selection on the Major Histocompatibility Complex. Crit. Rev. Immunol..

[56] Aeschlimann P.B., Häberli M.A., Reusch T.B.H., Boehm T., Milinski M. (2003). Female sticklebacks Gasterosteus aculeatus use self-reference to optimize MHC allele number during mate selection. Behav. Ecol. Sociobiol..

[57] Holling T.M., Schooten E., Elsen P.V.D. (2004). Function and regulation of MHC class II molecules in T-lymphocytes: Of mice and men. Hum. Immunol..

[58] Worley K., Collet J., Spurgin L.G., Cornwallis C., Pizzari T., Richardson D.S. (2010). MHC heterozygosity and survival in red junglefowl. Mol. Ecol..

[59] Takeshima S.N., Giovambattista G., Okimoto N., Matsumoto Y., Rogberg-Muñoz A., Acosta T.J., Onuma M., Aida Y. (2015). Characterization of bovine MHC class II DRB3 diversity in South American Holstein cattle populations. Tissue Antigens.

[60] Bjelland D., Weigel K., Vukasinovic N., Nkrumah J. (2013). Evaluation of inbreeding depression in Holstein cattle using whole-genome SNP markers and alternative measures of genomic inbreeding. J. Dairy Sci..

[61] Sieklicki M.D.F., Mulim H.A., Pinto L.F.B., Valloto A.A., Pedrosa V.B. (2020). Population structure and inbreeding of Holstein cattle in southern Brazil. Rev. Bras. Zootec..

[62] Silva A.R., Resende-Moreira L.C., Carvalho C., Lanes E.C.M., Ortiz-Vera M.P., Viana P.L., Jaffé R. (2020). Range-wide neutral and adaptive genetic structure of an endemic herb from Amazonian Savannas. AoB Plants.

[63] Gebremedhin B., Ficetola G.F., Naderi S., Rezaei H.-R., Maudet C., Rioux D., Luikart G., Flagstad Ø., Thuiller W., Taberlet P. (2009). Frontiers in identifying conservation units: From neutral markers to adaptive genetic variation. Anim. Conserv..

[64] Eckert C.G., Samis K.E., Lougheed S.C. (2008). Genetic variation across species’ geographical ranges: The central–marginal hypothesis and beyond. Mol. Ecol..

[65] Hoffmann A.A., Willi Y. (2008). Detecting genetic responses to environmental change. Nat. Rev. Genet..

